# Construction of a combined hypoxia and EMT index for head and neck squamous cell carcinoma

**DOI:** 10.3389/fcell.2022.961858

**Published:** 2022-08-15

**Authors:** Huan Li, Jun Wang, Lei He, Fengrui Zhang, Qingzhe Meng, Junhong Huang, Yahui Li, Rong Liu, Xinjie Yang, Jianhua Wei

**Affiliations:** ^1^ State Key Laboratory of Military Stomatology and National Clinical Research Center for Oral Diseases, and Shaanxi Clinical Research Center for Oral Diseases, Department of Oral and Maxillofacial Surgery, School of Stomatology, Fourth Military Medical University, Xi’an, China; ^2^ State Key Laboratory of Cancer Biology, Biotechnology Center, School of Pharmacy, Fourth Military Medical University, Xi’an, China

**Keywords:** HNSCC, EMT, immune, hypoxia, prognosis

## Abstract

**Objectives:** In head and neck squamous cell carcinoma (HNSCC), the interaction between epithelial-mesenchymal transformation (EMT) and hypoxia has been confirmed, and corresponding treatment methods have been investigated. Few studies have examined its combined effects and its potential clinical use, however. As a result, we developed a new scoring system based on EMT and hypoxia.

**Methods:** We combined 200 hypoxia-related genes with 1184 EMT-related genes and finally constructed a score risk model containing 14 characteristic factors named the comprehensive index of EMT and hypoxia (CIEH) by the Lasso-Cox regression and univariate Cox regression method, which is used to predict prognosis and to guide treatment planning in HNSCC patients. Furthermore, we examined HNSCC expression of CIEH-related genes using the human protein atlas database.

**Results:** Based on survival analysis results, CIEH value had a high prognostic value in HNSCC patients, a high CIEH value carries a poor prognostic significance in HNSCC. It is noteworthy that the CIEH value was correlated with tumor immune infiltration. Moreover, the CIEH had significant differences in age, stage, N, laterality, and peripheral nerve invasion, and that the CIEH could be an independent prognostic factor.

**Conclusions:** This study constructed a CIEH model containing 14 characteristic factors, including hypoxia-related genes and EMT genes, that may be able to serve as potential biomarkers for HNSCC. According to the 14 characteristic factors in the CIEH model, a diagnostic kit can be packaged in the future to evaluate the survival of patients before tumor surgery and guide the subsequent treatment plan.

## Introduction

Head and neck squamous cell carcinoma (HNSCC) is the 6th most common cancer in the world, and is a highly malignant tumor (Johnson et al., 2020). Surgery, adjuvant radiotherapy, and chemotherapy constitute the conventional treatment for HNSCC. Even though clinical treatment techniques and concepts continue to improve, patients with HNSCC still have a low 5-year survival rate (Cramer et al., 2019; Borel et al., 2020). The problems of long-term toxicity and organ function damage caused by current clinical treatment still exist. Thus, improving prognosis of HNSCC patients and studying molecular mechanisms of prognosis are urgently needed.

According to studies, changes in tumor microenvironment often lead to hypoxia of tumor cells in a variety of tumors including HNSCC, thus affecting tumor metabolism, proliferation, apoptosis, invasion and metastasis, immune escape and other biological processes (Schito and Semenza, 2016). It is important to note that studies have demonstrated that hypoxia-triggered angiogenesis causes immunosuppression, meaning hypoxia is a culprit of immunotherapy failure (Kopecka et al., 2021). Other studies have shown that PD-1 blockade resistance is associated with hypoxia in HNSCC (Zandberg et al., 2021). However, its mechanism is still unclear, and hypoxia-targeted therapy is unlikely to become a clinical treatment in the short term (Curtis et al., 2016).

EMT refers to a cellular process called epithelial-mesenchymal transition, in which epithelial cells develop mesenchymal characteristics along with fibroblast morphology and migration abilities, which play a key role in tumor metastasis (Singh et al., 2018). Studies have shown that the CCL21/CCR7 interaction enhances OSCC stemness and induces EMT (Singh et al., 2018). Some scholars have also found that LINC00460 promoted EMT in HNSCC cells *via* its ability to promote PRDX1 goes into the nucleus (Jiang et al., 2019). However, several recent studies indicate that hypoxia can change the morphology and characteristics of tumor cells and induce EMT to promote tumor cell metastasis (Zhang et al., 2013; Choi et al., 2017; Joseph et al., 2018). Some scholars have demonstrated that the lncRNA RP11-390F4.3 is activated by hypoxia/HIF-1α and contributes to tumor metastasis *via* multiple EMT-regulated factors (Peng et al., 2020). In HNSCC related studies, gefitinib resistance is caused by hypoxia, which promotes cell cycle progression and EMT (Yin et al., 2018).

Although hypoxia and EMT have been extensively studied, few studies have examined the potential clinical significance of their combined effects. In addition, there are quite a several bioinformatics studies to explore the factors affecting the prognosis of HNSCC, but these factors are often clinical factors and HPV status, and little consideration is given to the influence of tumor microenvironment, especially hypoxia, EMT and immune infiltration on the prognosis of cancer cells and patients (Zeng et al., 2020; Panuganti et al., 2021). Combining hypoxia and EMT gene data sets, this study screened HNSCC prognostic factors related to these two biological processes, constructed a comprehensive index of EMT and hypoxia (CIEH) model, and analyzed its effectiveness in multi-data sets. The differences between high and low CIEH values in immune infiltration, gene mutations, enrichment pathways and clinical features were explored. Based on the CIEH model, it may also be used in clinical practice as a prognostic tool for HNSCC patients in the future, and provide patients with individualized treatment options.

## Materials and methods

### Acquisition and preprocessing of data

From the MSigDB, 200 hypoxia-related genes (HRGs) have been downloaded (Liberzon et al., 2011) (https://www.gsea-msigdb.org/).

From the dbEMT database, 1184 genes related to EMT were downloaded (Zhao et al., 2019) (http://dbemt.bioinfo-minzhao.org/download.cgi).

TCGA-HNSCC expression profile data were downloaded from the UCSC Xena (https://xena.ucsc.edu/), and 20,530 identifiers × 566 samples were collected, of which 521 tumor samples were selected for further analysis. The downloaded data was normalized log2 (x+1) transformed RSEM normalized count, and the patient clinical data were downloaded on Xena as shown in Table 1.

**TABLE 1:** Transcriptome data set information.

Clinical data	TCGA-HNSCC Cohort (*n* = 521)		GEO65858 Corhort (*n* = 270)	
	n	%	n	%
Age, years				
>60	263	50.48	117	43.33
<=60	258	49.52	153	56.67
Gender				
Male	384	73.70	223	82.59
Female	137	26.30	47	17.41
T				
T1-2	186	35.70	115	42.59
T3-4	335	64.30	155	57.41
M				
M0	490	94.05	NA	NA
M1	6	1.15	NA	NA
N				
N0	243	46.64	94	34.81
N1-3	256	49.14	176	65.19
Alcohol				
YES	347	66.60	239	88.52
NO	163	31.29	31	11.48
Radiation				
YES	292	56.05	NA	NA
NO	159	30.52	NA	NA
Perineural invasion				
YES	169	32.44	NA	NA
NO	194	37.24	NA	NA
Stages				
I	20	3.84	18	6.67
II	97	18.62	37	13.70
III	105	20.15	37	13.70
IV	278	53.36	178	65.93

The microarray data GSE65858 of HNSCC was downloaded from GEO using R package GEO query, including 270 tumor patient transcriptome data and 31,330 probes. The expression data were log2-transformed and normalized using RSN. For the downloaded chip data, the limMA software package was used for standardization. The chip is the GPL10558 platform, which uses the annotation information of the platform to map probes to genes and remove empty probes. In the case that multiple probes corresponding to the same gene, the median was selected as the expression value of the gene, and the expression profile data of 22,001 gene symbols were obtained.

### Gene screening and visualization

The 200 HRGs and 1184 EMTs were mapped to the expression profiles of TCGA-HNSCC and GSE65858, and the genes detected in both profiles were screened for subsequent analysis. A total of 1048 genes were detected in both sets of data, including 180 HRGs and 919 EMTs. Venn diagram was drawn using R software package ggvenn. Circos diagram was drawn using RCircos to show the position of screening genes in chromosomes and their relative expression levels in TCGA and GSE65858.

### The development of the comprehensive index of EMT and hypoxia

An assessment of the prognostic relevance of HRGs and EMTs was carried out using univariate Cox regression. Twenty-six genes were found to be associated with prognosis. We constructed the LASSO Cox regression model by using R-packet glmnet to apply it to the model construction. During model construction, the coefficient of feature factors was estimated using the partial likelihood deviance with tenfold cross validation. Log (lambda) at −4.08 was found to be optimal. A total of 14 characteristic factors were obtained from the model. By using 14 genes screened by the LASSO model, the risk score was calculated under hypoxia and EMT conditions, and the formula was as follows:

$$Score=\sum_{i=1}^{n}\left(Genei\;\times \;Coefi\right)\;$$

According to the different risk scores of each patient sample, it could be categorized into high and low scoring groups, and PCA analysis was conducted. Next, we standardized the risk score value and constructed the comprehensive index of EMT and hypoxia (CIEH) model. Correlation between CIEH and model gene was analyzed using R software package cor. Here is how the CIEH was calculated:

CIEH=(Score-Min)/Max

### Analyze the functions of the comprehensive index of EMT and hypoxia

ROC and survival curves were drawn for the samples according to their CIEH values, which were divided into high and low CIEH samples. The 1-year and 3-year overall survival rates (OS) were plotted to show the difference in prognosis between the two groups. The patient scores were calculated according to the same formula, and the disease-free interval (DFI) data of the patients were analyzed to observe the prognosis of the two groups. The expression profiles of 22 groups of immune cells were downloaded from CIBERSORT (Newman et al., 2015) (http://cibersort.stanford.edu/), and an analysis of immune microenvironments in high and low CIEH groups was performed. Gene mutation data were downloaded from TCGA, and the mutation differences of HRGs and EMTs between high and low CIEH group were performed using the R package maftools.

To further analyze the differences between high-CIEH and low-CIEH samples, clusterprofiler was used for GSEA GO enrichment analysis. Visit the GSEA website to download GO biological processes. Significant enrichment results were screened according to corrected *p* values (*p* < 0.05). Based on the sample clinical information in the TCGA, the differences between low-CIEH and high-CIEH samples in other clinical prognostic factors were analyzed. The clinical information selected for the study included: Age, alcohol history, clinical stage, grade, M, N, radiotherapy, laterality and Perineural invasion. Combined with TCGA clinical information, a univariate and multivariate Cox regression analysis was performed on CIEH, and a nomogram was constructed.

### Immunohistochemical validation

The Human Protein Atlas provides immunohistochemical staining data (https://www.proteinatlas.org/). The HPA illustrates how proteins are distributed and their relative abundance in the human. Images of related characteristic proteins expressed in HNSCC and normal tissues were downloaded for analysis.

### Statistical analysis

Based on the coxph function of the R package, univariate Cox regression model was established based on survival information (*p* < 0.05). Based on LASSO Cox regression, the critical genes associated with HRGs and EMTs were identified. R-packet rms was used to draw nomogram based on survival information and clinical indicators to predict patients’ prognoses. R software was used to perform the statistical analyses (Version 3.6.3). All *P* values were considered two-tailed, and *p* < 0.05 was considered statistically significant.

## Results

### Prognostic associated hypoxia and epithelial-mesenchymal transformation-related genes

**FIGURE 1:**
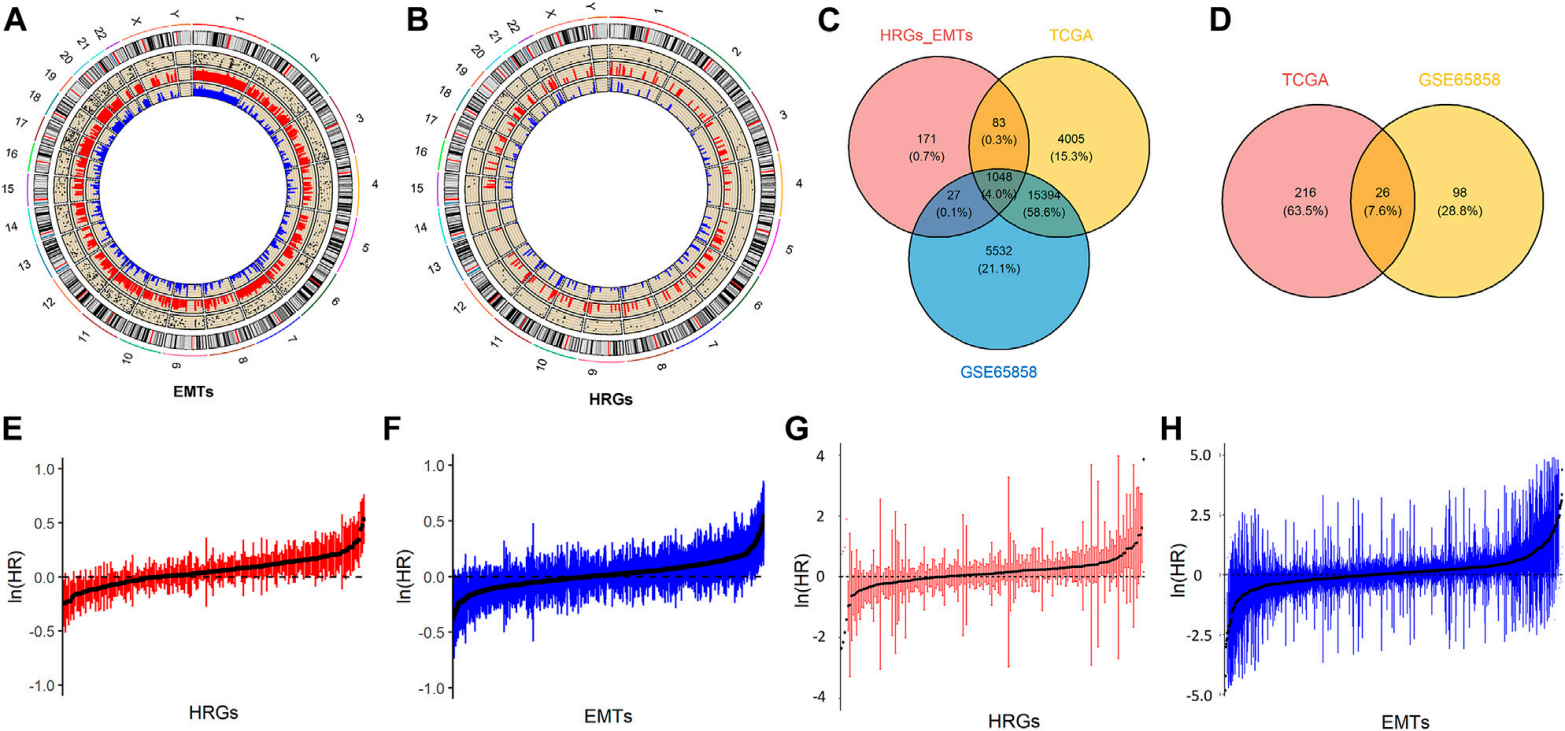
Prognostic associated HRGs and EMTs. **(A)**. Circos plot of EMTs, including the locations of candidate genes in the genome, and the relative expression levels in the TCGA and the GSE65858. **(B)**. Circos plot of HRGs, including the locations of candidate genes in the genome, and the relative expression levels in the TCGA and the GSE65858. **(C)**. Mapping results of HRGs and EMTs in TCGA and GSE65858 datasets. **(D)**. Prognostic factors were selected from TCGA and GSE65858 datasets. **(E,F)**. Visualization of HRGs and EMTs in TCGA dataset by univariate Cox regression analysis. **(G,H)**. Visualization of HRGs and EMTs in GSE65858 dataset by univariate Cox regression.

The data sets used in this study were TCGA-HNSCC and GSE65858, among which TCGA-HNSCC included 520 tumor patient samples and GSE65858 included 270 tumor patient samples. The downloaded 200 HRGs and 1184 EMTs were mapped to TCGA-HNSCC and GSE65858, and the genes detected in both sets of expression profiles were screened for subsequent analysis. A total of 1048 genes were detected in both sets of transcriptome data, including 180 HRGs and 919 EMTs. Figures 1A,B shows the locations of HRGs and EMTs on the genome and their expression information in TCGA and GSE56868. Figure 1C shows the mapping results of HRGs and EMTs in two transcriptome datasets.

TCGA-HNSCC and GSE65858 datasets were analyzed using univariate Cox regression to search for prognostic genes based on the 1048 genes above. A total of 242 prognostic genes were screened in the TCGA-HNSCC dataset and 124 prognostic genes were screened in the GSE65858 dataset, among which 26 prognostic genes were detected in both sets of data (Figure 1D). Figures 1E,F are the univariate Cox regression analysis results of HRGs and EMTs in the TCGA-HNSCC data set respectively. Similarly, the analysis results in GSE65858 are shown in Figures 1G,H.

### The creation of a comprehensive index for EMTs and hypoxia in head and neck squamous cell carcinoma

**FIGURE 2:**
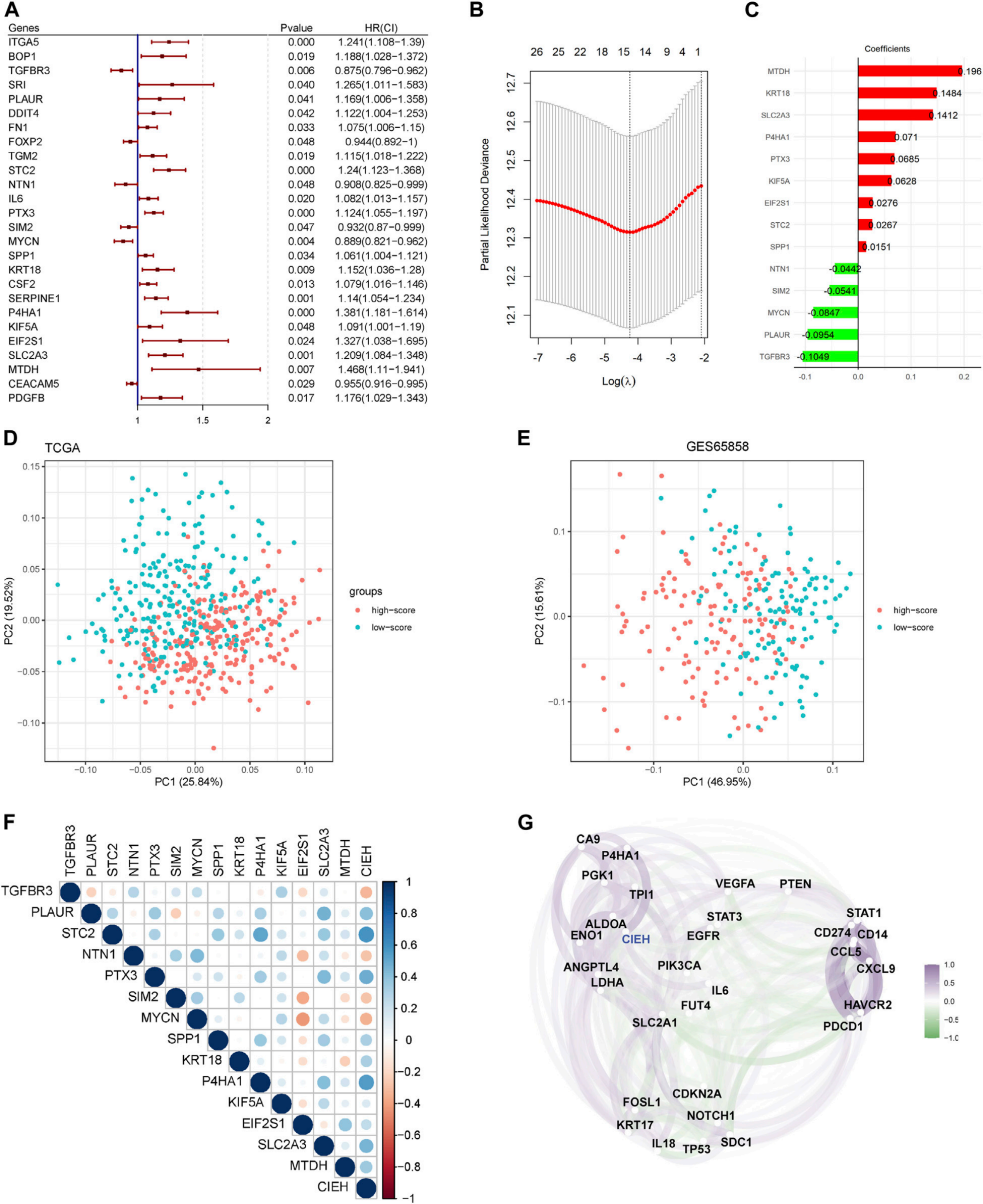
Predictive modeling and the CIEH. **(A)**. Univariate Cox regression analysis of twenty-six prognostic genes. **(B)**. Optimization parameters of lasso Cox model. **(C)**. Genes screened by Lasso Cox model and coefficient values of each gene. **(D)**. PCA analysis of high/low score groups in TCGA. **(E)**. Analyse of PCA for groups with high and low scores in GSE65858. **(F)**. Correlation between CIEH model and 14 prognostic factors. **(G)**. Correlation between CIEH and hypoxia-immune signature genes.

The 26 genes were obtained by univariate Cox regression analysis and further screened by the Lasso Cox regression model (Figures 2A,B). Finally, we obtained 14 prognostic characteristic factors, of which 9 were positively correlated with prognosis and 5 were negatively correlated with prognosis (Figure 2C). HRGs include PLAUR, STC2, SLC2A3, P4HA1 and KIF5A. EMTs include TGFBR3, PLAUR, MYCN, SIM2, NTM, SPP2, MTDH, KRT18, PTX3, EIF2S1, and STC2. Based on the coefficient in Figure 2C, we calculated the risk score, and a sample was divided into two groups based on its risk score value: High and Low. After that, a PCA analysis was conducted. Risk scores were significantly different between high and low risk groups in the TCGA-HNSCC and GSE65858 datasets (Figures 2D,E).

The risk score was standardized to construct the CIEH index. The correlation analysis between the CIEH value and 14 prognostic factors showed that CIEH value was highly correlated with it (Figure 2F). Moreover, the CIEH index was highly correlated with hypoxia-immune genes (Figure 2G). The hypoxia-immune signature genes were derived from the published data (PMID:31182433).

### Prognostic value of the comprehensive index of EMT and hypoxia

**FIGURE 3:**
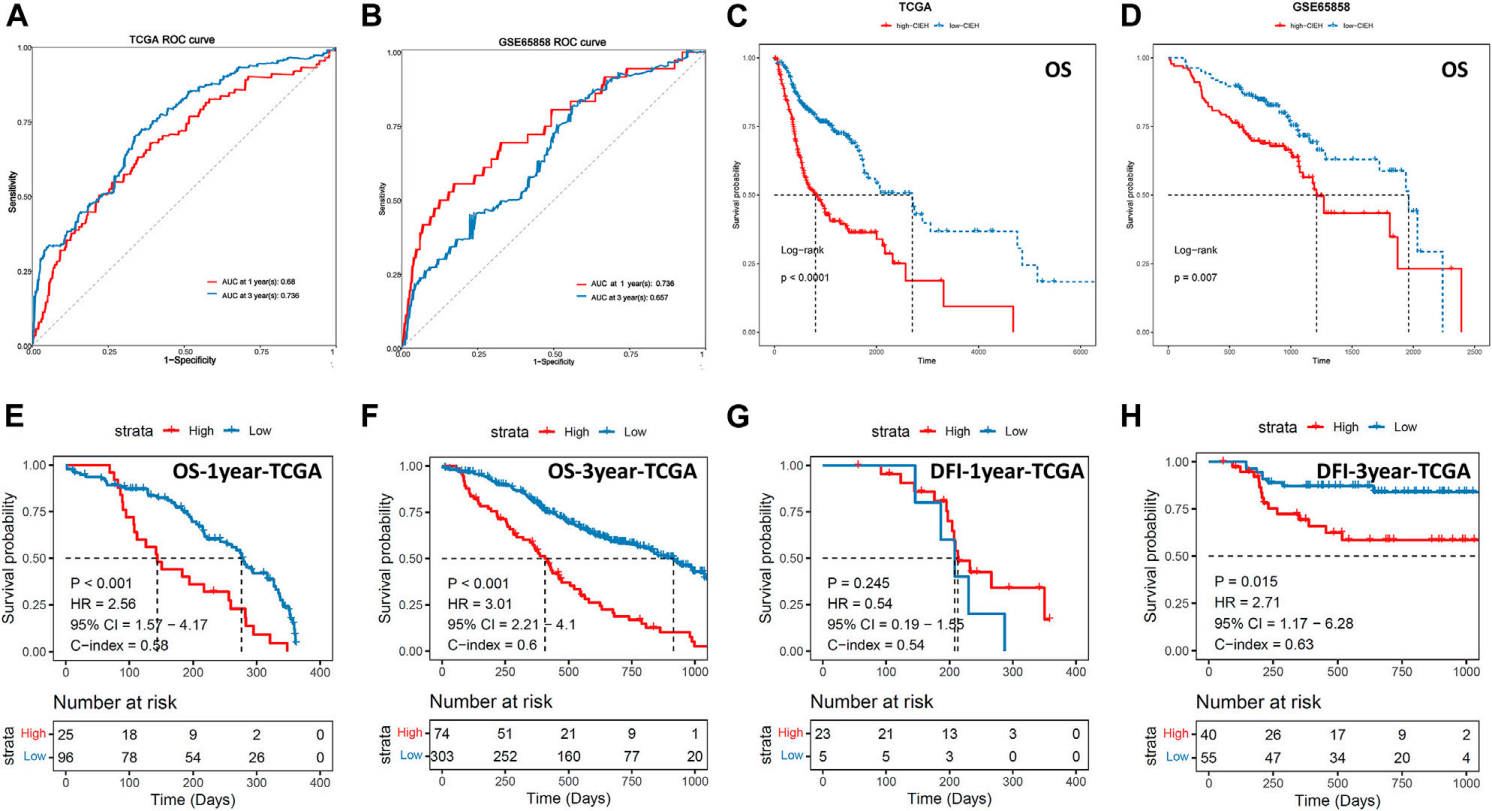
Effectiveness analysis of CIEH model prediction. **(A,B)**. ROC curves of CIEH values of TCGA and GSE65858 data sets. **(C,D)**. Overall survival analysis of TCGA and GSE65858 in high and low CIEH groups (*p* < 0.05). **(E)**. The 1-year overall survival analysis of TCGA (*p* < 0.05). **(F)**. The 3-year overall survival analysis of TCGA (*p* < 0.05). **(G)**. The 1-year disease-free interval (DFI) analysis of TCGA (*p* > 0.05). **(H)**. The 3-year disease-free interval (DFI) analysis of TCGA (*p* < 0.05).

The ROC curve of CIEH value showed that the AUC of 1-year and 3-year in TCGA dataset was 0.680 and 0.736, respectively (Figure 3A), and that of 1-year and 3-year in GSE65858 dataset was 0.736 and 0.657, respectively (Figure 3B). In addition, statistical analysis found that the prognosis of patients with high-CIEH differs significantly from those with low-CIEH, and low-CIEH patients had a relatively high overall survival (OS) rate (Figures 3C,D *p*< 0.05). Both 1-year and 3-year OS results showed that the CIEH value was significantly correlated with the prognosis of HNSCC patients (Figures 3E,F *p*< 0.05). The 3-year disease-free interval (DFI) data training set and OS results were similar, indicating that patients with high CIEH had a poor prognosis (Figure 3H *p*< 0.05). However, there was no significant difference in the results of 1-year DFI data training set (Figure 3G *p*> 0.05).

### Differences in immune function between high and low comprehensive index of EMT and hypoxia

**FIGURE 4:**
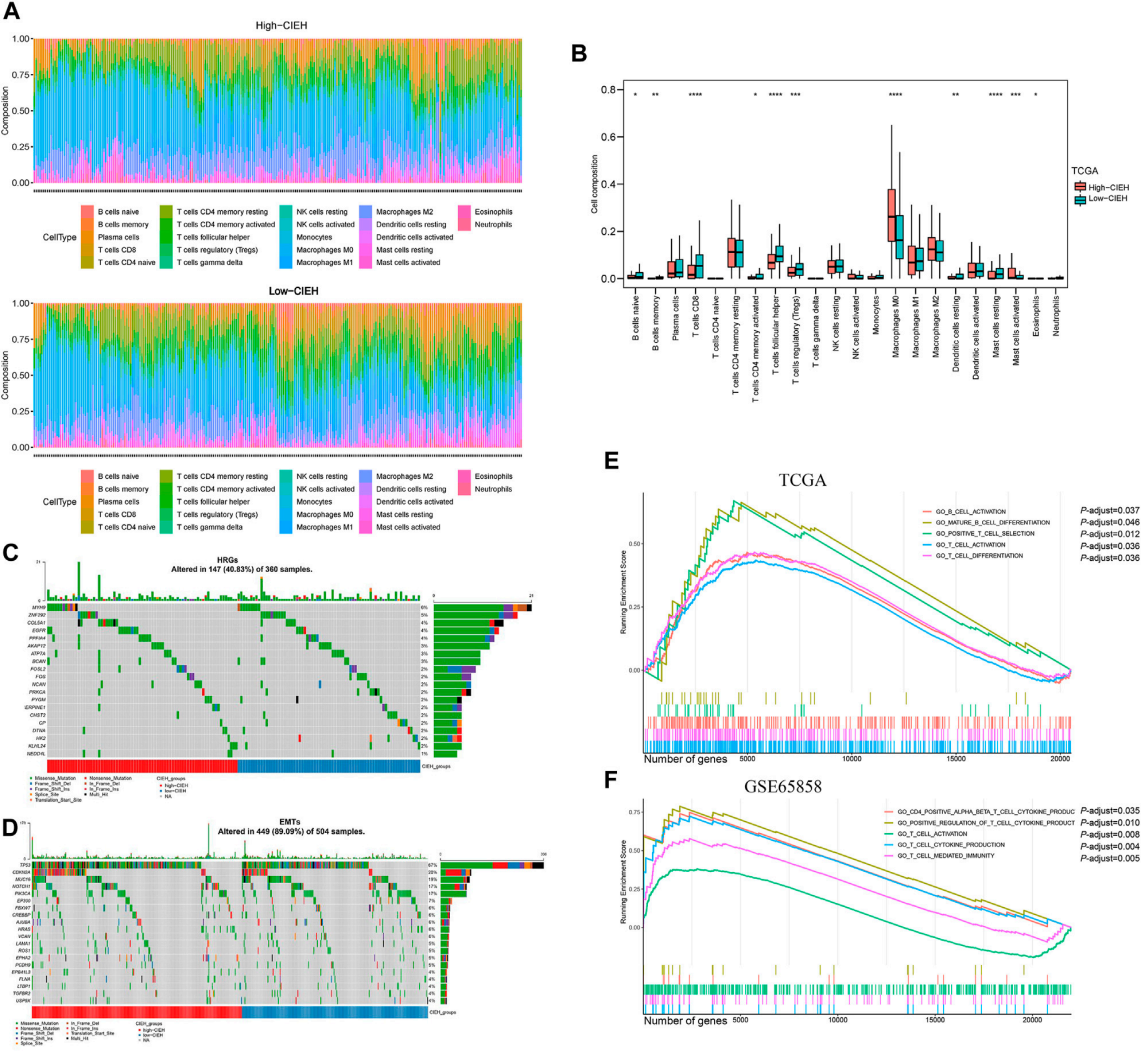
Immune function of the CIEH. CIBERSORT assessed the difference in immune infiltration between high and low comprehensive index of EMT and hypoxia (CIEH) groups. **(A)**. The difference between the proportion of immune cells in groups with a high and low CIEH. **(B)**. Comparing the immune cell composition of high and low CIEH groups. **(C,D)**. Mutation difference analysis of HRGs and EMTs between the high and low CIEH groups. **(E,F)**. GO enrichment between the high and low CIEH groups was analyzed in TCGA and GSE65858. The X scale is the number of genes. The short vertical line represents the position of the corresponding gene in the sequence list in the enrichment pathway.

In immune infiltration analysis (Figures 4A,B), the low-CIEH group had a higher number of B and T cells (*p*< 0.05). Compared with the low-CIEH group, the high-CIEH group had higher M0 macrophage content (*p*< 0.05). There was no significant difference in the number of plasma cells, NK cells, monocytes, macrophages M1, M2 and neutrophils between the two groups. Next, the mutation differences of HRGs and EMTs gene sets between high-CIEH and low-CIEH samples were further analyzed, and no significant mutation differences were found (Figures 4C,D).

To further study the functional differences between high and low CIEH groups, we conducted a GSEA enrichment analysis. A total of 321 biological process (BP) were enriched in the TCGA-HNSCC dataset and 799 BP were enriched in the GSE65858 dataset (Supplementary Tables S1, S2). It is worth mentioning that T-cell activation genes were significantly enriched in samples with low CIEH in both datasets (Figures 4E,F).

### Clinical characteristics of comprehensive index of EMT and hypoxia

**FIGURE 5:**
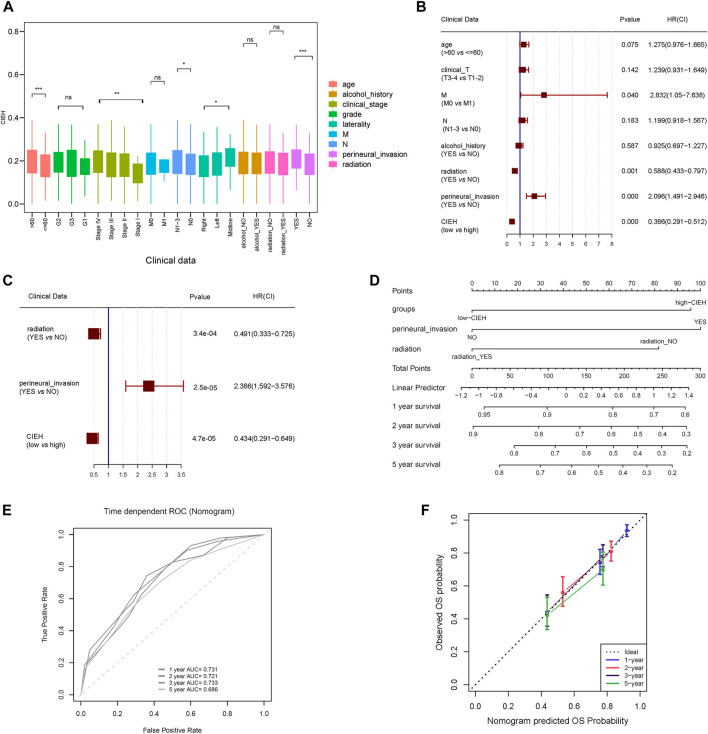
Clinical characteristics of CIEH. **(A)**. Difference analysis of CIEH model in clinical prognostic factors. **(B)**. Univariate Cox regression analysis of CIEH and other prognostic factors. **(C)**. Multivariate cox regression analysis of CIEH and other prognostic factors. **(D)**. Nomogram of CIEH combined with other prognostic factors to predict survival rate. **(E)**. ROC curve for predicting survival rate. 1-year AUC = 0.731; 2-year AUC = 0.721; 3-year AUC = 0.733; 5-year AUC = 0.686; **(F)**. Nomogram survival calibration curves at 1, 2, 3 and 5 years (C-indx = 0.698).

In the analysis of differences between CIEH value and clinical features, it was found that CIEH value had significant differences in age, stage, N, laterality and peripheral nerve invasion (Figure 5A). Univariate and multivariate Cox regression analysis revealed that radiation, Perineural invasion (PNI) and CIEH value were independent prognostic factors. The results showed that patients with radiation (YES) Perineural Invasion (NO) and CIEH (Low) had a good prognosis (Figures 5B,C). Nomogram was constructed for these three prognostic factors, and the results showed that the nomogram can predict the survival rates of HNSCC patients (Figure 5D). The ROC curve for predicting the HNSCC survival rate was Figure 5E. Figure 5F shows 1, 2, 3, and 5 years survival calibration curves. The concordance index (C-index) of the nomogram was 0.698, the AUC of 3-year survival was 0.733, and the AUC of 5-year survival was 0.686.

### Immunohistochemical

**FIGURE 6:**
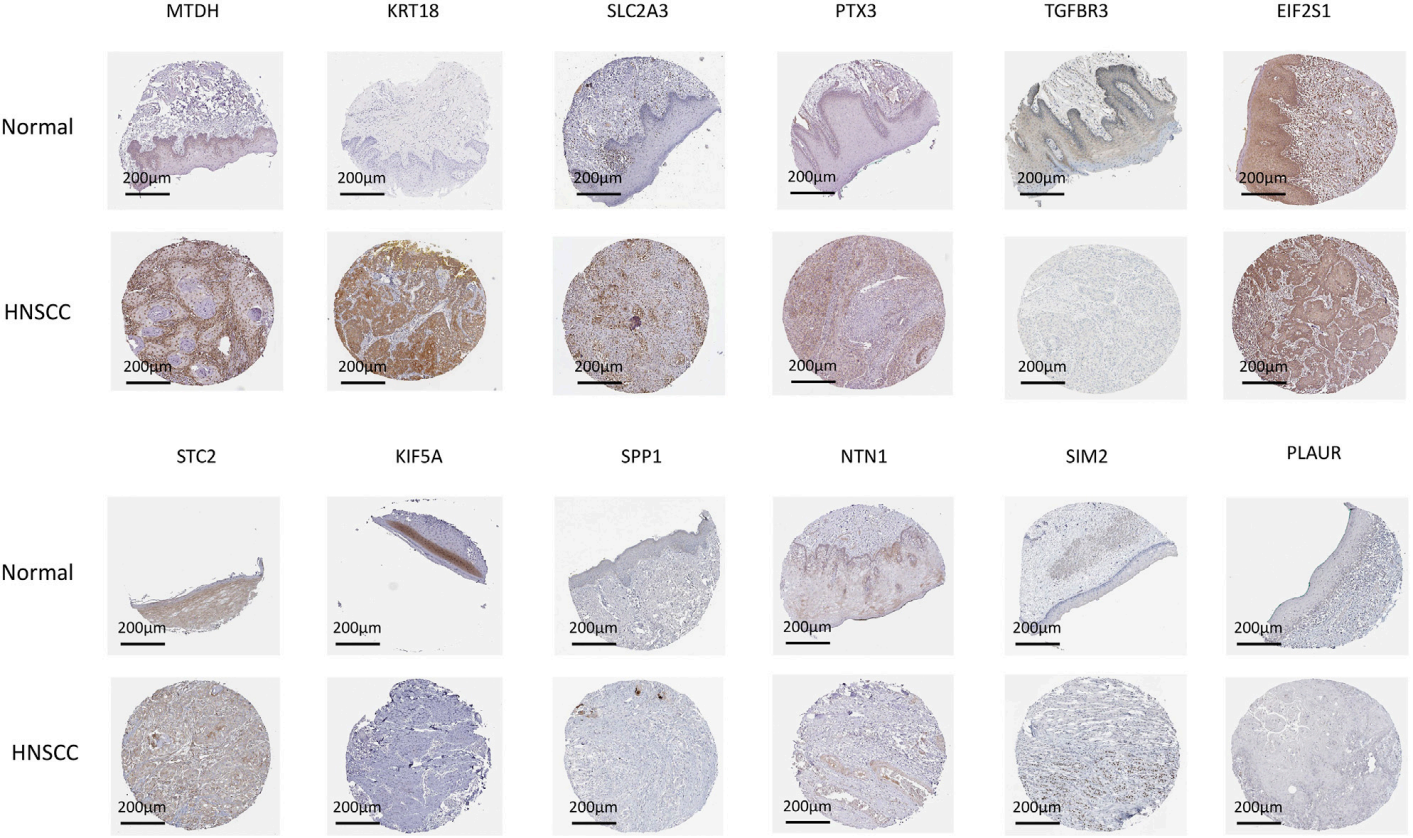
CIEH model immunohistochemistry validation. Using the HPA database, we analyzed the expression of related target proteins in normal tissues and HNSCC.

In order to analyze the expression of target proteins in HNSCC and normal tissue, we used the Human Protein Atlas (HPA) database (Figure 6). Compared to normal tissue, the expression of MTDH, KRT18, SLC2A3 and PTX3 were significantly higher in HNSCC tissue. Notably, a significant reduction in TGFBR3 expression was found in HNSCC in comparison with normal tissue. Furthermore, there were no significant differences in the expression of EIF2S1, STC2, KIF5A, SPP1, NTN1, SIM2 and PLAUR between HNSCC and normal tissue.

## Discussion

One of the most common malignant tumors, HNSCC is extremely malignant with a poor prognosis and a low 5-year survival rate. EMT and hypoxia are key features of HNSCC, as described in previous studies. It is emphasized that hypoxia is one of the important factors affecting EMT (Gonzalez-Gonzalez et al., 2021). It has also been reported that hypoxia is closely related to the tumor microenvironment, which can contribute to EMT and sometimes even the spread of cancer (Huang et al., 2018). Although there have been many studies on hypoxia or EMT, only a few studies have been performed on its combined effects and its potential clinical application.

A new study shows that a prognostic model based on hypoxia and immunity has been developed and validated in triple-negative breast cancer, which may lead to clinical improvements in hypoxia and immunotherapy (Zheng et al., 2020). Therefore, we combined 200 hypoxia-related genes and 1184 epithelial-mesenchymal transformation-related genes and finally constructed a score risk model containing 14 characteristic factors named comprehensive index of EMT and hypoxia (CIEH) by the Lasso-Cox regression method and univariate Cox regression, which is used to help guide the prognosis and clinical treatment of HNSCC patients. In a survival analysis model of HNSCC patients, the CIEH value can predict the prognosis of patients, and patients with high CIEH had a poorer prognosis. However, it should be emphasized that AUC of ROC analyses on 1-year or 3-year survival below 0.7, suggesting that the CIEH value was moderately accurate and should be further optimized in the future. According to the 14 characteristic factors in the CIEH model, a diagnostic kit can be packaged in the future to evaluate the survival of patients before tumor surgery and guide the subsequent treatment plan.

Numerous studies have indicated that immune cell infiltration can regulate tumor progression (Talmadge, 2011; Kim et al., 2020). HNSCC patients’ prognosis has also been related to immune cell infiltration (Schneider et al., 2018). In our manuscript, we found that the low-CIEH group had a higher proportion of T-cells and B-cells in immune infiltration compared with the high-CIEH group. Moreover, the T-cell activation-related genes were significantly enriched in low-CIEH samples in both TCGA and GSE65858. Based on the results, the comprehensive index of EMT and hypoxia was associated with tumor immune infiltration. In addition, we discovered that the CIEH had significant differences in age, stage, N, laterality, and peripheral nerve invasion, and that the CIEH could be an independent prognostic factor. Patients with low-CIEH have a good prognosis. In the future, we may collect a large number of clinical samples from HNSCC patients, carry out the detection of 14 characteristic factors related to CIEH, and conduct patient follow-up to verify the validity of this model through single-center or multi-center clinical trials.

In addition, we carefully analyzed the expression of CIEH-related genes in HNSCC using HPA database. According to the results, MTDH, KRT18, SLC2A3 and PTX3 were highly expressed in HNSCC tissues, while TGFBR3 was low. Prognostic factors included MTDH, KRT18, SLC2A3 and PTX3, while TGFBR3 exhibited a negative correlation with the prognosis. The expression of MTDH, KRT18, SLC2A3, PTX3 and TGFBR3 in HNSCC was correlated with prognosis. Some researchers have found that miR-98 serves as a suppressor in HNSCC progression *via* targeting oncogene MTDH (Tan et al., 2017). Studies have also shown that KRT18 plays a pivotal role in colorectal cancer progression, suggesting that the protein may be a therapeutic target for improving the prognosis of patients with colorectal cancer (Zhang et al., 2019). The SLC2A3 gene is also associated with a shorter survival time and a worse quality of life when it comes to colorectal cancer patients (Kim et al., 2019). Some studies have shown that Pentraxin-3 (PTX3) is an immune modulator thought to play a role in tumor angiogenesis, proliferation, and escape of tumors (Netti et al., 2020). Results in LSCC showed that circ_0042666 regulates miR-223/TGFBR3 expression in laryngeal squamous cell carcinoma cells (Wei et al., 2019). These CIEH-related genes are associated with tumor progression or patient prognosis in many tumors, so it is very important to study their synergistic effect in HNSCC in the future.

In conclusion, this study constructed a CIEH model containing 14 characteristic factors including hypoxia-related genes and epithelial-mesenchymal transformation genes, which may serve as potential biomarkers for HNSCC. In the future, the CIEH model may be applied to clinical treatment planning for HNSCC patients to predict their prognosis and facilitate the selection of appropriate treatment options.

## Data Availability

The datasets presented in this study can be found in online repositories. The names of the repository/repositories and accession number(s) can be found in the article/Supplementary Material
